# Review of preventative HIV vaccine clinical trials in South Africa

**DOI:** 10.1007/s00705-020-04777-2

**Published:** 2020-08-14

**Authors:** Fatima Laher, Linda-Gail Bekker, Nigel Garrett, Erica M. Lazarus, Glenda E. Gray

**Affiliations:** 1 Perinatal HIV Research Unit, Faculty of Health Sciences, University of the Witwatersrand, Johannesburg, South Africa; 2 The Desmond Tutu HIV Foundation, University of Cape Town, Cape Town, South Africa; 3 Centre for the AIDS Programme of Research in South Africa, Durban, South Africa; 4 Department of Public Health Medicine, School of Nursing and Public Health, University of KwaZulu-Natal, Durban, South Africa; 5 South African Medical Research Council, Cape Town, South Africa

## Abstract

New HIV infections continue relentlessly in southern Africa, demonstrating the need for a vaccine to prevent HIV subtype C. In South Africa, the country with the highest number of new infections annually, HIV vaccine research has been ongoing since 2003 with collaborative public-private-philanthropic partnerships. So far, 21 clinical trials have been conducted in South Africa, investigating seven viral vectors, three DNA plasmids, four envelope proteins, five adjuvants and three monoclonal antibodies. Active vaccine candidates have spanned subtypes A, B, C, E and multi-subtype mosaic sequences. All were well tolerated. Four concepts were investigated for efficacy: rAd5-gag/pol/nef showed increased HIV acquisition in males, subtype C ALVAC/gp120/MF59 showed no preventative efficacy, and the trials for the VRC01 monoclonal antibody and Ad26.Mos4.HIV/subtype C gp140/ aluminum phosphate are ongoing. Future trials are planned with DNA/viral vector plus protein combinations in concert with pre-exposure prophylaxis, and sequential immunization studies with transmitted/founder HIV envelope to induce broadly neutralizing antibodies. Finally, passive immunization trials are underway to build on the experience with VRC01, including single and combination antibody trials with an antibody derived from a subtype-C-infected South African donor. Future consideration should be given to the evaluation of novel strategies, for example, inactivated-whole-virus vaccines.

## Introduction

The geographical disparity of the annual 1.7 million new human immunodeficiency virus (HIV) infections [1] substantiates that southern Africa is most in need of a preventative vaccine. Subtype C predominates in southern Africa [2], where a third of the world’s new infections occur. In 2018, South Africa (n = 240,000) and Mozambique (n = 150,000) accounted for the highest numbers of new infections, almost a quarter of global infections [1].

In this millennium, greater attention is being given to developing HIV vaccines in South Africa with efforts spanning from the characterization of subtype C viral genetics with the purpose of informing vaccine constructs, to the first human HIV vaccine clinical trials in the country [3]. Unlike most vaccine research trials in Africa, which are funded by private industry [4], HIV vaccine research has been funded largely by the United States (US) government through the National Institutes of Health. More recently, the Bill and Melinda Gates Foundation and the European and Developing Countries Clinical Trials Partnership have also committed funding. The South African Medical Research Council invested in the development of subtype C vaccines under the auspices of the South African AIDS Vaccine Initiative (SAAVI) from 1999 [3]. Local investment by African governments for HIV vaccine research has been limited, there are few vaccinology training programmes, and there is a lack of vaccine design and manufacturing capability, all of which compound the vaccine development gap in Africa [5]. Owing to the requirement for vaccine development expertise and a research infrastructure to conduct HIV vaccine trials, the enterprise has been collaborative [6]. Partners for HIV vaccine research in South Africa have included product developers such as AlphaVax, Merck, Sanofi, GlaxoSmithKline, Novartis and Janssen, as well as consortia such as SAAVI, the International AIDS Vaccine Initiative (IAVI), and the HIV Vaccines Trials Network (HVTN) and African universities and non-governmental organizations.

Our literature review is a narrative of preventative HIV vaccine clinical trials conducted in South Africa. Although the topic is not within the scope of this review, we note that South Africa has also conducted clinical research into therapeutic HIV vaccines, including a tat vaccine that, in phase 2 testing, has demonstrated CD4^+^ T-cell recovery and viral reservoir reduction [7].

In our review, we find that 21 clinical trials have been conducted in South Africa from 2003 until the time of writing (Table 1). Most were conducted with adult participants (20/21), one with infants (1/21), and none with adolescents below the age of 18 years old. About half of the trials (11/21) were phase I trials. Four concepts were investigated for efficacy, two of which are ongoing. Only one regimen reached phase IIb-III, but it was not efficacious. Of the trials conducted in adults, three-quarters (15/20) were conducted with participants who were at low risk of HIV acquisition, and a fifth (4/20) with individuals at risk, predominantly young heterosexual adults. One trial recruited in low- and medium-risk categories (1/20). Overall, seven viral vectors have been studied with various inserts of gag, protease, pol, env, nef, reverse transcriptase and tat genes from subtypes A, B, C, E and mosaic sequences (Table 2). Three DNA plasmids have been investigated with various inserts of gag, pol, env, nef, reverse transcriptase and tat genes from subtypes A, B, and C (Table 3). Four envelope proteins originating from subtypes B, C and E, and five adjuvants have been tested (Table 4). Three monoclonal antibodies are currently being investigated. Many trials (12/21) have enrolled participants in countries outside South Africa as well, especially other African countries and the US.

**Table 1:** Preventative HIV vaccine clinical trials conducted in South Africa

Year started [Ref]	Clinical trial (phase)	Investigational products (M = month)	Population: number enrolled, risk level, countries	Outcomes
2003 [13]	IAVI 011 (I)	HIVA.MVA vector vaccine at M0, 2	N = 81 low-risk adults, SA, Kenya, Switzerland, UK	Generally safe. Common but tolerated local reactions. Rare responses of T cells expressing gamma interferon on ELISPOT. No further development warranted.
2003 [14]	HVTN 040 (I)	AVX101, a VEE vector vaccine at M0, 1, 3	N = 48 low-risk adults, SA, Botswana, US	Vaccinations stopped after product manufacturing issues. Well tolerated. Dose-dependent Ab responses to gag and VEE but marginal T-cell response.
2005 [16]	HVTN 050 (I)	MRKAd5 HIV-1 gag, an Ad5 vector vaccine, at M0, 1, 6.5	N = 360 low-risk adults, global	Well tolerated. Immunogenic.
2005 [15]	IAVI A002 (II)	tgAAC09, a rAAV2 vector vaccine, at M0, 6 or M0, 12	N = 91 low-risk adults, SA, Uganda, Zambia	Generally safe, well-tolerated. HIV-specific T cells expressing gamma interferon by ELISPOT in a quarter of vaccine recipients. No further development warranted.
2005 [14]	HVTN 059 (I)	AVX101, a VEE vector vaccine, at M0, 1, 3	N = 84 low-risk adults, SA, Botswana, US	Vaccinations stopped after contract manufacturer documentation issues. Well tolerated.
2006 [40]	HVTN 204 (II)	VRC-HIVDNA-016-00-VP, a DNA plasmid vaccine, at M0, 1, 2 plus rAd5, an Ad5 vector vaccine, at M6	N = 480 low- to medium-risk adults, SA	Well-tolerated. Induced polyfunctional CD4^+^ and CD8^+^ T-cells and multi-subtype anti-Env binding antibodies.
2007 [18]	HVTN 503 (IIb)	MRKAd5 HIV-1 gag/pol/nef, an Ad5 vector vaccine, at M0, 1, 6.5	N = 801 at-risk adults, SA	Enrolment stopped after step trial futility results. Well-tolerated. No efficacy. Most produced T-cell response to subtypes B, C. No viral setpoint lowering in HIV-infected. Increased HIV risk in vaccine recipients.
2009 [41]	HVTN 073 and 073 Extension (I)	SAAVI DNA-C2, a DNA plasmid vaccine, at M0, 1, 2. MVA-C, an MVA vector vaccine, at M4,5. In the extension, Novartis subtype C gp140, a protein vaccine, at M24, 27	N = 48 low-risk adults, SA	Generally safe, well-tolerated. CD4^+^ T cell responses mostly to env tier 1 neutralizing Abs present only after protein boost, strongest against subtype C isolates. Binding antibody responses initially weak, then 100% after protein boost and maintained over 6 months.
2011 [42]	HVTN 086 (I)	SAAVI DNA-C2, a DNA plasmid vaccine; MVA-C, an MVA vector vaccine; Novartis subtype C gp140, a protein vaccine	N = 184 low-risk adults, SA	Generally safe, well-tolerated. Sequential protein-boost regimens induced strong peak neutralizing and binding antibody responses but decayed with time. Protein-boost regimens induced T-cell responses. MVA/protein prime boost regimen induced strongest neutralizing antibody and T cell responses.
2011 [38]	HVTN 091 (I)	Ad26.ENVA.01, an Ad26 vector vaccine; Ad35-ENV, an Ad35 vector vaccine. Homologous and heterologous regimens, M0, 3 or M0, 6	N = 218 low-risk adults, SA	Generally safe, well-tolerated. Humoral and cellular immune responses in most participants, not affected by pre-existing Ad26- or Ad35-neutralizing antibodies. In homologous and heterologous regimens, the second vaccination increased EnvA antibody titers. Heterologous Ad26–Ad35 elicited significantly higher EnvA antibody titers than Ad35–Ad26. Modest T-cell responses.
2013 [31]	HVTN 097 (Ib)	ALVAC-HIV(vCP1521), a canarypox vector vaccine at M0, 1, 3, 6 and AIDSVAX B/E, a protein vaccine at M3, 6	N = 100 low-risk adults, SA	Generally safe, well-tolerated. At peak, higher cellular and antibody responses than RV144 vaccinees. Env-specific IgG and CD4+ Env responses declined significantly over time.
2014 [39]	HIV-V-A004 (I-IIa)	Ad26.Mos.HIV, an Ad26 vector vaccine; gp140, a protein vaccine; MVA, a vector vaccine	N = 393 low-risk adults, US, Rwanda, SA, Thailand, Uganda	Generally safe, well-tolerated. Ad26 prime/Ad26+gp140 boost was the most immunogenic regimen, eliciting Env-specific binding antibody (100%), ADCP (80%) and T-cell responses (83%).
2015 [29]	HVTN 100 (I-II)	Subtype C ALVAC-HIV (vCP2438), a canarypox vector vaccine, at M0, 1, 3, 6, 12 and bivalent subtype C gp120/MF59, a protein vaccine, at M3, 6, 12	N = 252 low-risk adults, SA	Generally safe, well-tolerated. Most produced IgG and T-cell responses. Month 12 booster restored immune responses and slowed response decay compared to month 6 vaccination.
2015 [55]	IMPAACT P1112 (I)	VRC01, VRC01LS or VRC07-523LS, monoclonal antibodies in single or multiple doses	N = 40 HIV-exposed infants, SA, Zimbabwe, US	Single and multiple doses of subcutaneous VRC01 were generally safe and well tolerated in infants.
2016 [59]	HVTN 703 (IIb)	VRC01, a monoclonal antibody, at M0, 2, 4, 6, 8, 10, 12, 14, 16, 18	N = 1926 at-risk women, SA, Malawi, Mozambique, Zambia, Zimbabwe	Infusions concluded. Follow-up ongoing.
2016 [12]	HVTN 111 (I)	DNA-HIV-PT123, a DNA plasmid vaccine, and MF59- adjuvanted subtype C gp120, a protein vaccine	N = 132 low-risk adults, SA, Tanzania, Zambia	Generally safe, well-tolerated. DNA and protein co-administration associated with increased V1V2 antibody response rate. DNA by electroporation elicited higher CD4^+^ T-cell response rates to HIV envelope protein than by needle/syringe in prime/boost regimen, but not in coadministration regimen.
2016 [35]	HVTN 702 (IIb-III)	Subtype C ALVAC-HIV (vCP2438), a canarypox vector vaccine, at M0, 1, 3, 6, 12, 18 and bivalent subtype C gp120/MF59, a protein vaccine, at M3, 6, 12, 18.	N = 5407 at-risk adults, SA	Vaccinations stopped Feb 2020 for no efficacy. No safety concerns.
2017 [35]	HVTN 107 (I-IIa)	Subtype C ALVAC-HIV (vCP2438), a vector vaccine; bivalent subtype C gp120, a protein vaccine, alone, with MF59 or aluminium hydroxide adjuvant	N = 132 low-risk adults, SA, Zimbabwe, Mozambique	Results expected in 2020.
2017 [43]	HVTN 108 (I-IIa)	DNA-HIV-PT123, a DNA plasmid vaccine, and MF59 or AS01_B_ adjuvanted subtype C gp120, a protein vaccine	N = 334 low-risk adults, SA, US	Acceptable safety profiles. At peak, all groups had high IgG response rates to gp140, gp120, V1V2. AS01_B_-adjuvanted groups showed improved CD4^+^ response rates and magnitudes.
2018 [35]	HVTN 705 (IIb)	Ad26.Mos4.HIV, an Ad26 vector vaccine, at M0, 3, 6, 12 and aluminum-phosphate-adjuvanted subtype C gp140, a protein vaccine, at M6,12	N = 2637 at-risk women, SA, Malawi, Mozambique, Zambia, Zimbabwe	Enrolment completed. Follow-up ongoing.
2019 [60]	CAPRISA 012A (I)	VRC07-523LS and/or PGT121, monoclonal antibodies, at M0 or 2 timepoints 3, 4 or 6 months apart	N = 45 low-risk women, South Africa	Injections concluded. Follow-up ongoing.

**Table 2:** Viral vectors and gene inserts used in HIV preventative vaccines in South Africa [13–16, 18, 29, 31, 35, 38–42]

Viral vector	SA trial use	HIV antigen gene insert: subtype_strain_ origin						
		gag	protease	RT	pol	env	nef	gag/RT/tat/nef
Adeno-associated virus serotype 2	IAVI A002	C_DU422_	C_DU422_	C_DU422_				
Adenovirus serotype 5 (Ad5)	HVTN 050	B_near consensus_						
Ad5	HVTN 204	B_HXB2-NL4-3_			B_HXB2-NL4-3_	A_92rw020_, B_HXB2/Bal_, C_97ZA012_		
Ad5	HVTN 503	B_CAM-1_			B_IIIB_		B_JR-FL_	
Ad26	HVTN 705	Mosaic 1&2			Mosaic 1&2	Mosaic 1&2S		
Ad26	HVTN 091					gp140-A_92rw020_		
Ad26	HIV-V-A004	Mosaic 1&2			Mosaic 1&2	Mosaic 1		
Ad35	HVTN 091					gp140-A_01TZA173_		
Canarypox	HVTN 097	B_LAI_	B_LAI_			gp41-B_LAI_, env-E_TH023_		
Canarypox	HVTN 100, HVTN 107, HVTN 702	B_LAI_	B_LAI_			gp120-C_ZM96_, gp41 TM-B_LAI_		
Modified Vaccinia Ankara (MVA)	IAVI 011	A_consensus_, CD8 T-cell epitope			CD8 T-cell epitopes	CD8 T-cell epitopes	CD8 T-cell epitopes	
MVA	HVTN 073, HVTN 086					gp150CT-C_Du151_		C_Du422,Du151,Consensus Du422/Du151,Du151_
MVA	HIV-V-A004	Mosaic 1&2			Mosaic 1&2	Mosaic 1&2		
Venezuelan equine encephalitis (VEE) virus	HVTN 040, HVTN 059	C_DU422_

**Table 3:** DNA plasmids and gene inserts used in HIV preventative vaccines in South Africa [12, 40–43]

DNA plasmid	SA trial use	HIV antigen gene: subtype_strain_ origin						
		gag	protease	RT	pol	env	nef	gag/RT/tat/nef
Clade C DNA-HIV-PT123	HVTN 108, HVTN 111	C_96ZM651_			C_CN54_	gp140-C_96ZM651_	C_CN54_	
SAAVI DNA-C2	HVTN 073, HVTN 086					C_Du151_		C_Du422,Du151,Consensus Du422/Du151,Du151_
VRC-HIVDNA-016-00-VP	HVTN 204	B_HXB2, NL4-3, NY5/BRU_			B_HXB2, NL4-3, NY5/BRU_	A_92rw020_, B_HXB2/BaL_, C_97ZA012_	B_HXB2, NL4-3, NY5/BRU_

**Table 4:** HIV subunit proteins used in HIV preventative vaccines in South Africa [12, 29, 31, 35, 39, 41–43]

Protein	Structure	SA trial use		HIV envelope subtype_strain_ origin
		Adjuvant	Trial	
AIDSVAX B/E	Monomeric gp120	Aluminum hydroxide	HVTN 097	B_MN_, E_A244_
Bivalent subtype C gp120	Monomeric gp120	MF59	HVTN 100, HVTN 107, HVTN 108 HVTN 111	C_TV1.C, 1086.C_
		Aluminium hydroxide	HVTN 107	
		AS01_B_	HVTN 108	
		No adjuvant	HVTN 107	
Clade C gp140	Trimeric gp140	Aluminium phosphate	HIV-V-A004, HVTN 705	C_CZA97012_
Novartis subtype C gp140	Oligomeric gp140	MF59	HVTN 073 Extension, HVTN 086	C_TV1_

All 18 active vaccination trials have employed the prime-boost strategy of immunization, which administers antigens at the initial priming dose/s and the booster dose/s. Seven trials investigated homologous prime-boost strategies, defined as administering the same antigen, in the same antigen delivery format, during the prime and the booster doses. Twelve trials investigated heterologous prime-boost strategies, defined as when the prime and the booster are the same antigen in different antigen delivery formats, or when the primes and boosters are dissimilar antigens [8].

To date, preventative HIV vaccine clinical trials in South Africa have vaccinated with either HIV components or with HIV antibodies, and we review their findings through this thematic framework.

## Vaccinating with HIV components

Most HIV vaccine trials in South Africa have employed the strategy of presenting antigens to the immune system, either indirectly, for example through a recombinant vector vaccine or DNA plasmid vaccine with inserts encoding for the expression of the antigen/s of choice, or directly with an HIV subunit vaccine consisting of the antigen/s of choice.

### Vectors: recombinant viral vector and DNA plasmid vaccines

Vectors are bacteria, viruses or nucleic acids that have been modified to incorporate gene/s encoding antigen/s of choice [9]. Vector vaccines elicit intracellular gene transcription, and the subsequent antigen presentation induces predominantly cellular immune responses. Viral and DNA plasmid vectors have been investigated clinically in South Africa [9]. Compared to bacterial vectors, viral vectors are generally easier to bio-engineer and cheaper to develop, and they produce robust adaptive immune responses [9]. Moreover, there is unlikely to be pre-existing immunity to vectors based on viruses that do not typically infect humans, minimising the possibility of attenuation of immune reponses [10]. DNA plasmid vaccines further reduce interference from an immune response directed at a viral or bacterial vector [10]. Their small size also allows for novel administration routes, such as needle-free injection systems like Biojector, although this may increase costs [11]. Preliminary data suggests that CD4^+^ T-cell response rates may be increased by Biojector administration of a DNA plasmid prime plus a protein boost [12]. Additionally, DNA plasmids are cheap and easy to manufacture. To date in South Africa, all HIV DNA plasmid vaccines have been investigated in combination with other vaccines (Section 2.3).

The first five HIV vaccine trials that were conducted in South Africa (2003-2005) administered recombinant vectors only. The vectors, all viral, had inserts of various combinations of HIV genes (most frequently gag) isolated from subtypes A, B or C. IAVI 011 evaluated a 2-dose regimen of a modified vaccinia Ankara (MVA) vector expressing HIV-1 subtype A gag p24 and p17 antigens at two dose levels via various routes: low dose subcutaneous and intramuscular and mid dose (subcutaneous, intramuscular and intradermal). Although there were no serious adverse events, the vaccine did cause some severe local reactions. Based on a low rate and durability of immune responses seen in the concurrent IAVI 010 trial, which included the same vaccine candidate and a higher dose level, further development of the candidate vaccine was considered futile, so enrolment and vaccinations in IAVI 011 were halted [13]. HVTN 040 and HVTN 059 assessed a Venezuelan equine encephalitis virus (VEEV)-based replicon vaccine, AVX101, expressing the DU422 isolate subtype C gag gene [14]. The vaccine was well tolerated and demonstrated dose-dependent antibody responses with modest T-cell responses. However, due to vaccine stability issues, HVTN 040 was halted prior to completion of all dose levels, and HVTN 059, which had been implemented to obtain additional safety and immunogenicity data following improvements in manufacturing methods of AVX101, was also halted after issues were identified with the contract manufacturer’s documentation and environmental monitoring program [14]. The IAVI A002 trial of tgAAC09, a recombinant vaccine based on adeno-associated virus serotype 2 encoding HIV-1 subtype C Gag, protease, and part of reverse transcriptase, was safe and well tolerated. However, it produced low to moderate antibody and cellular responses, possibly owing to low inflammatory responses to the vector. No further development was warranted [15]. The HVTN 050 trial found that the MRKAd5 HIV-1 subtype B gag vaccine was safe and immunogenic [16].

HVTN 502 (conducted in the Americas) and HVTN 503 (conducted in South Africa) tested a similar vaccine to HVTN 050, but with additional pol and nef gene insertions. The vaccine induced immune responses, which were shown to be influenced by the following variables: female sex predicted higher subtype C antigen immune responses and being overweight and heavy alcohol use predicted reduced responses [17]. However, the immunogenicity generated from the vaccine did not translate to efficacy [18, 19]. HVTN 503 was the first vaccine efficacy trial conducted in South Africa, but enrolment was halted when HVTN 502 demonstrated futility [18]. Increased HIV acquisition was observed in male vaccine recipients in HVTN 503, irrespective of serological adenovirus serotype 5 (Ad5) status and circumcision status, prompting discontinuation of further Ad5-based vaccine research in southern Africa [20]. (In the US, however, HVTN 505, which included an Ad5 booster in addition to a DNA plasmid prime, was initiated after HVTN 502 and HVTN 503 in Ad5 seronegative male participants and demonstrated neither enhancement nor efficacy [21].) The reasons behind the enhancement seen in HVTN 502 and 503 are not yet fully understood and are complex and multi-factorial. One of the possible reasons was underpinned by the quandary that although vaccine-induced immune responses may include T cell proliferation, including long-lived memory T cells, CD4 T cells are known to be HIV targets. One *in vitro* study demonstrated that the Ad5 vector rendered HIV susceptibility through multiple mechanisms: the Ad5 vector induced CD4 T-cell proliferation, and those cells also had high surface expression of CCR5 and CXCR4, the chemokine coreceptors necessary for HIV entry. In comparison, ALVAC induced CD8 T-cell proliferation and less chemokine coreceptor expression [22].

### HIV subunit vaccines: protein vaccines

HIV subunit vaccines present an antigen directly to the immune system. Major advantages of HIV subunit vaccines are the lack of live HIV, stability, and low cost. However, there are complexities with protein vaccines. Proteins require administration with adjuvants for immunogenicity (though the generation of immune responses is not the same thing as efficacy for preventing HIV acquisition), and the optimal protein-adjuvant combination cannot be predicted but must be determined by clinical trials [23]. Moreover, viral proteins and synthetic peptides used as vaccines may not necessarily structurally resemble the conformation of the actual folded HIV protein, and so these vaccines may induce antibodies different from the ones that would be necessary to bind to the virus itself [23].

All nine trials in South Africa using the subunit vaccination approach have administered a variation of the HIV envelope protein: gp120 or gp140. Generally, peptide vaccines have had limited success in preventing infections with any pathogen, including HIV [24]. Even before HIV vaccine trials were initiated in South Africa, it had been established that subunit envelope protein vaccines alone were not efficacious in preventing HIV infection [25]. Therefore, in South Africa, protein vaccines have been investigated only in combinations with other HIV vaccine candidates, and only after the 2009 announcement in Thailand that the RV144 regimen of a vector and adjuvanted protein had partial efficacy [26]. Of the two combination regimen trials with protein vaccines in South Africa, HVTN 702 has not shown efficacy, and HVTN 705 is ongoing.

### Combination regimens

Subunit vaccines are thought to induce antibody responses. DNA plasmid vaccines, recombinant live vectors, and live attenuated viruses are thought to stimulate cellular responses. Combination regimens carry the theoretical advantage of stimulating multiple parts of the immune system and bypassing the induction of anti-vector immunity seen during homologous prime-boost vaccinations with live vectors [27]. However, there are cost, regulatory, and logistic drawbacks of complex regimens. Vaccine-induced immunity may be affected by pre-immunity against vaccine vectors, number of doses, dosing intervals, immunization routes, and adjuvants [28].

#### Viral vector plus protein

##### RV144 follow-on trials in South Africa

The Thai RV144 trial evaluated vaccines that had been designed to match HIV-1 strains commonly circulating in Thailand at the time [26]. The 6-month regimen was that of two canarypox primes of ALVAC-HIV (vCP1521), expressing subtype E env and subtype B gag and pro, followed by two ALVAC-HIV + AIDSVAX subtype B/E gp120 protein boosts [26]. The regimen demonstrated modest efficacy, which was statistically significant in a modified intention-to-treat analysis, but not in the per-protocol analysis [26].

After results were announced, the P5 (Pox-Protein Public Private Partnership) tailored the RV144 regimen to subtype C HIV strains. Adaptations of the RV144 vaccine regimen to South Africa included the use of the 96ZM651 gp120 env insert (subtype C) rather than the TH023 gp120 env insert (subtype E) used in RV144, inclusion of two subtype C gp120 Env proteins (TV1.C and 1086.C) in boost vaccinations (rather than subtype B and E proteins used in RV144), dose adjustments of the vector and protein, use of the MF59 instead of alum adjuvant, and the addition of boosters at month 12 and, in HVTN 702, month 18 [29]. The TV1.C and 1086.C gp120 Env proteins comprising bivalent subtype C gp120 were selected from candidate subtype C gp120 proteins according to an algorithm incorporating many factors. These included genetic relatedness to regional HIV strains, CCR5 binding, capacity in animal studies to elicit key epitope-specific antibodies, and percent monomer expression [30].

South Africa conducted a phase I trial, HVTN 097, of the partially efficacious RV144 regimen, ALVAC plus alum-adjuvanted AIDSVAX B/E [31]. The results were compared to those of another phase I trial, HVTN 100, which investigated the subtype C ALVAC and MF59-adjuvanted gp120 regimen. The immunological profiles of the two regimens were different, and it was found that the selected vaccine inserts contributed to these differences [32]. The RV144/HVTN 097 regimen induced greater magnitude and breadth of V2 responses than the HVTN 100/HVTN 702 regimen, but the HVTN 100/HVTN 702 regimen induced higher gp120 and ADCC responses [31]. However, when the subtype B/E and subtype C vaccines were compared in terms of the IgG antibody response against the V1V2 loop region of the 1086C HIV envelope, a lower proportion of participants responded to the subtype C vaccine. The difference in responses between the RV144/HVTN 097 and HVTN 100/HVTN 702 regimens suggest that the choice of viral sequences inserted into prime-boost vaccine regimens influences the elicitation of V2-specific antibodies [32]. V2-specific IgG antibodies had been observed as one of the correlates of protection in the RV144 vaccine trial in Thailand. In the subtype B/E vaccines, the inclusion of the A244 antigen efficiently induced V2 antibody responses, but the antigens in the subtype C vaccines were not as effective in accomplishing V2-directed responses [32].

The difference in the results between RV144, which showed partial efficacy, and HVTN 702, which showed none [33], has placed the field at a crossroad. Evidently, region-specific trials of the same strategy are necessary. The detection of immune responses in an early-phase trial amongst low-risk individuals is not a guarantee or even a suggestion that efficacy would manifest in advanced-phase testing. The interaction between molecules of the immune system and HIV occur within a complex context that is not yet even fully elucidated [24].

Besides the differences in the vaccines, there are also stark differences between Thailand and South Africa, and the most obvious is the circulating level of HIV. In 2018, South Africa had >37 times the number of incident HIV cases compared to Thailand [1]. HVTN 702 enrolled a population at high risk of HIV in South Africa and found no efficacy [33]. In the Thai trial, vaccine efficacy amongst those categorized as high risk was minimal (3.7%; 95%CI -72.7 to 46.3), while efficacy was highest in the medium-risk group 47.6% (95%CI -6.0 to 74.0) [26]. In the HVTN 702 trial, similar to the ECHO contraception trial released the previous year, HIV incidence was recorded at around 4%, and this was despite making the optimal standard of prevention care accessible [33, 34]. This highlights the ongoing gap in the HIV prevention market for at-risk populations in South Africa, and shows that the need for an efficacious vaccine remains urgent.

Might the HVTN 702 regimen have been efficacious if it had selected a different vector (DNA plasmid instead of canarypox), adjuvant (alum or AS01_B_ instead of MF59), or gp120 protein dose (40 mcg or 600 mcg instead of 200 mcg)? Or might a different dosing strategy (co-administration instead of prime-boost) or administration method (Biojector instead of needle-syringe) make a difference? These alternative subtype C vaccine approaches were investigated in four phase I/IIa trials (HVTN 107, HVTN 108, HVTN 111 and HVTN 120), in parallel with the trials that had hoped to develop subtype C ALVAC/gp120/MF59 for licensure (HVTN 100 and HVTN 702). Comparing the induction of immune correlates of protection across these phase I/IIa trials will provide guidance on an approach that might yield better efficacy outcomes than HVTN 702.

HVTN 107 investigated immune responses generated by MF59 versus aluminium hydroxide (the adjuvant used in the RV144 regimen) versus no adjuvant on the background regimen of subtype C ALVAC/gp120 [35]. Results are expected in 2020.

##### Viral vector plus protein: mosaic regimens

HIV sequences vary worldwide, posing a challenge to HIV vaccine development. A systematic review and global survey found the following frequencies of HIV-1 infection by subtype: C (46.6%), B (12.1%), A (10.3%), CRF02_AG (7.7%), CRF01_AE (5.3%), G (4.6%), D (2.7%), F, H, J, K combined (0.9%), other circulating recombinant forms (CRFs) (3.7%), and unique recombinant forms (6.1%) [36]. Moreover, sequence diversity is evolving quickly toward an increasing proportion of recombinants [36]. Polyvalent ‘mosaic’ antigens are proposed as a solution to optimize coverage.

The HVTN 705 trial is currently investigating, in sub-Saharan African women, the efficacy of synthetic mosaic antigens designed by computational optimization (“*in silico*”) from fragments derived from natural sequences [37]. The vector being used in HVTN 705, adenovirus serotype 26 (Ad26), had first been trialled in HVTN 091, demonstrating a good safety profile and induction of CD4^+^ T-cell responses to the HIV envelope [38]. The mosaic concept was first investigated in South Africa in the phase I trial HIV-V-A004, which is demonstrating durable immune responses in a long-term extension study [39].

There are notable differences between the Ad26/gp140 (HVTN 705) and ALVAC/gp120 (HVTN 702) regimens [35, 39]. First, the vaccine constructs are substantially different: the vectors (Ad26 versus canarypox), the inserts (synthetic mosaic antigens versus subtype B and C antigens from natural strains), the protein (monomeric gp120 versus trimeric gp140), and the adjuvant (aluminum phosphate versus MF59) [35, 39]. Second, the vaccine-induced immunogenicity profiles are distinct: Ad26/gp140 induces higher CD8 responses to gp140 and gp41 and more durable antibody and cellular responses (up to 3 years versus up to 6 months), while ALVAC/gp120 induces higher V2, including IgG3 V2, responses [32, 39]. In non-human primate studies, the Ad26/gp140 regimen had demonstrated protection from experimental challenge, identifying the following correlates of reduced acquisition: non-neutralizing binding antibodies to HIV envelope, ELISPOT T-cell responses expressing gamma interferon, and antibody-dependent cellular phagocytosis (ADCP) [39]. These correlates were not the same as the correlates of protection identified in the human RV144 trial [35, 39].

#### DNA plasmid plus vector

HVTN 204 was a phase II placebo-controlled trial in South Africa and the Americas that tested the safety and immunogenicity of a VRC multi-subtype DNA-HIV prime and rAd5-HIV boost regimen with subtype A, B and C env, gag-pol and nef immunogens [40]. The DNA-HIV vaccine (VRC-HIVDNA-016-00-VP) was composed of six DNA plasmids in equal concentrations that encode gag, pol, and nef from subtype B and HIV-1 Env glycoproteins from subtypes A, B and C (Table 3). The replication-defective recombinant Ad5-HIV vaccine (VRC-HIVADV014-00-VP, rAd5) contained a mixture of four rAd5 vectors encoding the HIV-1 Gag-Pol polyprotein from subtype B and HIV-1 Env glycoproteins from subtypes A, B and C matching the DNA Env components (Table 2). Participants were evenly randomized to receive either DNA at 0, 1 and 2 months followed by rAd5 at 6 months, or placebo. At month 12, T-cell responses were observed in 70.8% of vaccine recipients, most frequently to gag and env [40]. The vaccine induced a high frequency (83.7%–94.6%) of binding antibody responses to consensus group M and subtype A, B and C gp140 Env oligomers [40]. Antigen-specific T-cell responses were reduced in Ad5-antibody-seropositive individuals [40]. Despite demonstrating immunogenicity, further clinical evaluation of the Ad5 vector used in HVTN 205 did not continue in South Africa based on the results of HVTN 503 [18]. The HVTN 204 regimen was tested further in the US with seronegative men in HVTN 505 but was found to be futile [21].

SAAVI and the NIH designed subtype C vaccine constructs. The combination of the SAAVI DNA plasmid plus the SAAVI MVA vector was tested in the HVTN 073 trial [41]. HVTN 073 demonstrated modest immunogenicity induced by SAAVI DNA-C2, a multigene subtype C DNA vaccine with two DNA plasmids, and with SAAVI MVA-C, a multigene subtype C recombinant modified vaccinia Ankara vaccine [41]. In its extension phase, HVTN 073 administered a late boost with adjuvanted protein (Section 2.3.3).

Given the non-efficacious result of the HVTN 505 trial of DNA plasmid/rAd5 in 2013 in the Americas [21], no further investigations of the DNA plasmid plus vector strategy have been conducted in South Africa.

#### DNA plasmid plus vector plus protein

The HVTN 073 phase I safety and immunogenicity trial investigated the SAAVI HIV-1 subtype C DNA vaccine encoding Gag-RT-Tat-Nef and gp150, boosted with MVA expressing matched antigens (Tables 2 and 3). A total of 48 participants were randomized to receive three doses of SAAVI DNA-C2 (months 0, 1, and 2) followed by two doses of SAAVI MVA-C at months 4 and 5 or placebo [41]. After the release of the RV144 trial findings, it was decided to add a protein boost with HIV-1 subtype C V2-deleted gp140 with MF59 to the regimen. Two years after vaccination, 27 participants were therefore re-randomized to receive two doses of gp140/MF59, 3 months apart [41]. The DNA-MVA regimen elicited CD4^+^ T-cell and CD8^+^ T-cell responses in 74% and 32% of the participants, respectively, and the protein boost increased CD4^+^ T-cell responses to 87% [41]. All participants developed tier 1 HIV-1C neutralizing antibody responses as well as durable Env-binding antibodies [41]. Overall, the HIV-1 subtype C DNA-MVA vaccine regimen showed promising cellular immunogenicity. Boosting with gp140/MF59 enhanced levels of binding and neutralizing antibodies as well as CD4^+^ T-cell responses to the HIV-1 envelope [41].

The safety and immunogenicity of SAAVI DNA-C2, SAAVI MVA-C and Novartis V2-deleted subtype C gp140 with the MF59 adjuvant was further evaluated in the HVTN 086 trial [42]. At three South African sites, 184 HIV-uninfected participants were randomized (1:1:1:1) to one of four vaccine regimens: MVA prime, sequential gp140 protein boost (M/M/P/P); concurrent MVA/gp140 (MP/MP); DNA prime, sequential MVA boost (D/D/M/M); DNA prime, concurrent MVA/gp140 boost (D/D/MP/MP); or placebo [42]. The M/M/P/P and D/D/MP/MP regimens induced the strongest peak neutralizing and binding antibody responses and the greatest CD4^+^ T-cell responses to Env [42]. The MVA, but not DNA, prime contributed to the humoral and cellular immune responses [42]. The D/D/M/M regimen was poorly immunogenic overall but did induce modest CD4^+^ T-cell responses to Gag and Pol. CD8^+^ T-cell responses to any antigen were low for all regimens [42]. Overall, the SAAVI DNA-C2, SAAVI MVA-C and Novartis gp140 with MF59 adjuvant in various combinations were safe and induced neutralizing and binding antibodies and cellular immune responses [42]. Sequential immunization with gp140 boosted immune responses primed by MVA or DNA. The best overall immune responses were seen with the M/M/P/P regimen [42]. SAAVI did not pursue this vaccine regimen in light of the advancement of the P5 vaccine programme in South Africa.

#### DNA plasmid plus protein

The P5 vaccine programme researched the combination of DNA plasmid with a protein in two early-phase trials, HVTN 111 and HVTN 108 [35]. HVTN 111 compared the safety and immunogenicity of a subtype C DNA plasmid prime followed by DNA plasmid plus subtype C gp120 protein boost, with DNA/protein co-administration injected intramuscularly via either needle/syringe or a needle-free Biojector [12]. Interestingly, DNA and protein co-administration was associated with an increased V1/V2 antibody response rate, one of the correlates of decreased HIV-1 infection risk identified in RV144 [12]. DNA administration by Biojector elicited significantly higher CD4^+^ T-cell response rates to HIV envelope protein than administration by needle/syringe in the prime/boost regimen, but not in the co-administration regimen [12].

HVTN 108 evaluated the safety and immunogenicity of the DNA plasmid vaccine with different combinations of subtype C protein and adjuvant (MF59 or AS01_B_). In this phase I/IIa trial, 334 HIV-uninfected participants from South Africa and the US were randomized into seven intervention groups or placebo [43]. The regimens included DNA prime at months 0 and 1 with DNA/protein/adjuvant boosts at months 3 and 6, DNA/protein/adjuvant co-administration at months 0, 1 and 6, and only low-dose protein/AS01_B_ at M0, 1 and 6. DNA/protein/adjuvant combinations demonstrated acceptable safety profiles but more reactogenicity events in the AS01_B_ groups [43]. With the exception of DNA prime with low-dose protein and AS01_B_ boost, all intervention groups showed high IgG response rates to gp120 and gp140 and robust responses to V1V2 [43]. The AS01_B_-adjuvanted groups showed the highest CD4^+^ T cell responses [43]. Overall, the DNA/protein co-administration with AS01_B_ seemed to be the most promising regimen for further development.

### Where to from here for an active vaccine for South Africa?

There are plans to investigate further the combination of DNA plasmids with adjuvanted proteins with or without vectors. PrEPVacc will examine a novel strategy using pre-exposure prophylaxis during the vaccination period [44]. It is planned as a phase IIb trial with 1668 at-risk adults in South Africa, Uganda, United Republic of Tanzania, and Mozambique. Participants will first be randomized to an HIV vaccine regimen: DNA-HIV-PT123+AIDSVAX®/BE or DNA-HIV-PT123+CN54gp140/MPLA and MVA-CMDR+CN54gp140/MPLA or placebo. Thereafter, participants will be randomized a second time to tenofovir alafenamide/emtricitabine (Descovy) or tenofovir disoproxil fumarate/emtricitabine (Truvada) pre-exposure chemoprophylaxis [44].

Will we need to consider yet other vaccination strategies? Many subunit vaccines have now been investigated, but does this crossroad present the opportunity to consider a different inactivated vaccine strategy? For example, the inactivated-whole-virus vaccine strategy is used in the inactivated polio vaccine, rabies vaccine, and influenza vaccine. Although a subtype C inactivated whole vaccine is not yet in trials, recently in Canada, the subtype B NL4-3 strain of HIV was genetically modified and inactivated chemically and by radiation [45]. It was proven safe and immunogenic in a phase I trial, amongst HIV-infected individuals on anti-retroviral treatment [45]. Immune responses against subtype C HIV were not described. The inactivated-whole-virus vaccine strategy would need to be proven both safe and immunogenic in HIV-uninfected individuals to progress.

Active vaccination strategies to prevent HIV have focused on adult populations in South Africa. However, there is a gap in the development of an active vaccine to prevent HIV transmission through breast milk [46]. To address this need, HVTN 135 has been approved by regulatory authorities and is expected to start in 2020. This phase I trial pioneers two innovative vaccine concepts. The first is attempting to induce broadly neutralizing antibodies against HIV through active vaccination. Infant immune systems have unique characteristics that may facilitate the development of vaccine-induced broadly neutralizing antibody responses [47]. The second is the investigation of antigens derived from transmitted/founder viruses instead of from isolates in chronically infected individuals. Although it is not the most common variant in the donor, a single transmitted/founder virus establishes most cases of HIV infection [48]. HVTN 135 proposes to investigate CH505TF gp120, which is an envelope protein derived from transmitted/founder subtype C HIV in an acutely infected Malawian individual. CH505TF binds with high affinity to a broadly neutralizing antibody lineage and may contribute to an ultimate strategy of sequential immunization.

## Antibodies

After acquisition, HIV quickly proliferates and diversifies, creating evolving targets for antibody responses. From as early as one year after infection, but generally between 2 and 3 years, 10-30% of infected individuals generate antibodies that neutralize most HIV strains, called broadly neutralizing antibodies (bnAbs) [49]. Natural bnAb generation requires affinity maturation of B cells, which is the process of B cells being exposed repeatedly to antigen and producing antibodies with sequentially higher binding strengths between the HIV epitope and the antibody [49]. Affinity maturation may occur as successive antibodies induced by a series of antigenic variants appearing during chronic infection, but also as antibodies induced by the same viral antigen [50]. BnAbs have been noted to be structurally different to other antibodies [51]. More recently, bnAbs have been investigated for their HIV prevention potential [52]. Passive immunization has long been employed for other infectious diseases. No antigens are given in this strategy.

### Infused antibody

VRC01, a broadly neutralising monoclonal antibody, is the passive immunization agent in the most advanced phase of testing [52]. An IgG1 antibody, VRC01, was isolated from a subtype-B-infected long-term non-progressor. HVTN 703, which is an efficacy trial of VRC01, aims to prove the concept that a passively administered broadly neutralising antibody can cause reduction in HIV acquisition in African women [52]. In this study, VRC01 is administered intravenously over 30 minutes every two months. As such, it would carry pragmatic challenges to roll out on a mass scale, especially in constrained health systems [53]. Ultimately, if the concept of antibody-mediated prevention is proven, more-practical delivery platforms would need to be employed.

### Subcutaneous injection of antibody

Subcutaneous injection of monoclonal antibodies is being researched as a more pragmatic delivery platform. CAPRISA 012A is currently investigating the safety and pharmacokinetics of PGT121 and VRC07-523LS. PGT121 is a recombinant human IgG1 antibody isolated from a subtype-A-infected elite neutralizer who was not an elite controller [54]. VRC07-523LS is an engineered antibody based on VRC01.

Monoclonal antibodies are also being investigated to augment the elimination of vertical transmission of HIV. Passive vaccination of infants has the potential to prevent breast milk transmission of HIV. IMPAACT P1112 was the first trial to investigate monoclonal antibodies against HIV in infants. VRC01, VRC01LS and VRC07-523LS were administered subcutaneously to HIV-exposed infants. VRC01 demonstrated a good safety profile when administered as either a single dose or in multiple doses [55].

### Antibody combinations

Monoclonal antibody combinations are in the pipeline. IAVI C100 is a phase I-II trial that proposes to investigate a combination of two monoclonal antibodies, 3BNC117-LS-J and 10-1074-LS-J, amongst 225 low-risk adults in South Africa, the US, Kenya, Rwanda, and Uganda [56]. Enrolment is planned to begin in 2020. 3BNC117-LS-J is based on 3BNC117, an antibody isolated from an American elite HIV controller who has subtype B HIV [57]. 10-1074-LS-J is based on 10-1074, an antibody isolated from the same donor from whom PGT121 was isolated [54].

### Where to from here for a passive vaccine for South Africa?

CAPRISA 012B plans to investigate CAP256V2LS, an engineered antibody based on a neutralising antibody isolated from the subtype-C-infected donor CAP256 [58]. The clinical development pathway is aimed toward combination administration.

It is possible that, in the future, identification of efficacious broadly neutralizing antibodies could inform the design of an active vaccine by mapping epitopes on the HIV envelope.

## Conclusions

A vaccine effective against subtype C is important for epidemic control. HIV vaccine research has been robust in South Africa for 17 years. Clinical investigation has focused largely on active vaccination candidates based on many HIV subtypes and strains, but more recently, passive immunization has also been investigated. Many active vaccine regimens have shown safety and even immune responses, but few have reached efficacy testing. Of those that have, the immune responses have not been protective against HIV infection, and there is still no licensed vaccine.

It would be prudent to learn from the non-efficacy result of HVTN 702 to inform development of vaccines aimed at inducing correlates of protection as observed in RV144. Comparing the immune correlates of protection across P5 trials may help identify optimized strategies for subtype C vaccination. Amongst the available early-phase trial results, co-administration of DNA plasmid and AS01_B_-adjuvanted protein is promising.

In all likelihood, however, the field will need to investigate novel antigens and strategies. Particularly if the HVTN 703 results demonstrate that bnAb infusion can prevent HIV in sub-Saharan Africa, the novel strategy of sequential immunization with bnAb lineage antigens will be a promising active vaccination approach to induce bnAbs.

Antigens derived from transmitted/founder viruses are also an intriguing new pathway for the field. There are only a few different transmitted/founder viruses and envelope amino acid sequences. Possibly, the difference in efficacy outcome between RV144 and HVTN 702 may be because RV144 vaccine inserts included antigens that represented a more homogenous local viral diversity. Subtype C viruses are more heterogeneous, so using vaccine antigens derived from transmitted/founder viruses may be advantageous.

However, it is possible that the field may need to move toward approaches not reliant on the direct or indirect presentation of select antigens to the immune system. Although the inactivated whole-virus vaccine strategy has long been shunned by the HIV vaccine field, there is now some evidence that it may be worth pursuing, and though not within the scope of this paper, it is now plausible to consider the completely novel strategy of human genetic engineering to delete HIV co-receptor genes and mimic natural immunity.

Alongside the critical need to develop HIV vaccines for adult populations, pursuing the elimination of vertical transmission through vaccine approaches is also an imperative for South Africa. Furthermore, despite the lack of HIV vaccine research with adolescents thus far, the high burden of acquisition in young girls and young men who have sex with men highlights the need for future vaccine research with adolescent populations in South Africa.
